# A scalable model of vegetation transitions using deep neural networks

**DOI:** 10.1111/2041-210X.13171

**Published:** 2019-03-21

**Authors:** Werner Rammer, Rupert Seidl

**Affiliations:** ^1^ Department of Forest‐ and Soil Sciences Institute of Silviculture University of Natural Resources and Life Sciences (BOKU) Vienna Vienna Austria

**Keywords:** deep neural networks, ecological forecasting, simulation modelling, state and transition modelling, upscaling, vegetation dynamics, vegetation transitions

## Abstract

1. In times of rapid global change, anticipating vegetation changes and assessing their impacts is of key relevance to managers and policy makers. Yet, predicting vegetation dynamics often suffers from an inherent scale mismatch, with abundant data and process understanding being available at a fine spatial grain, but the relevance for decision‐making is increasing with spatial extent.
2. We present a novel approach for scaling vegetation dynamics (SVD), using deep learning to predict vegetation transitions. Vegetation is discretized into a large number (10^3^–10^6^) of potential states based on its structure, composition and functioning. Transition probabilities between states are estimated via a deep neural network (DNN) trained on observed or simulated vegetation transitions in combination with environmental variables. The impact of vegetation transitions on important ecological indicators is quantified by probabilistically linking attributes such as carbon storage and biodiversity to vegetation states.
3. Here, we describe the SVD approach and present results of applying the framework in a meta‐modelling context. We trained a DNN using simulations of a process‐based forest landscape model for a complex mountain forest landscape under different climate scenarios. Subsequently, we evaluated the ability of SVD to project long‐term vegetation dynamics and the resulting changes in forest carbon storage and biodiversity. SVD captured spatial (e.g. elevational gradients) and temporal (e.g. species succession) patterns of vegetation dynamics well, and responded realistically to changing environmental conditions. In addition, we tested the computational efficiency of the approach, highlighting the utility of SVD for country‐ to continental scale applications.
4. SVD is the—to our knowledge—first vegetation model harnessing deep neural networks. The approach has high predictive accuracy and is able to generalize well beyond training data. SVD was designed to run on widely available input data (e.g. vegetation states defined from remote sensing, gridded global climate datasets) and exceeds the computational performance of currently available highly optimized landscape models by three to four orders of magnitude. We conclude that SVD is a promising approach for combining detailed process knowledge on fine‐grained ecosystem processes with the increasingly available big ecological datasets for improved large‐scale projections of vegetation dynamics.

## 1 INTRODUCTION

Terrestrial vegetation is of crucial importance for human well‐being, providing a wide variety of ecosystem services to society (MEA, 2005) and forming the backbone of a large number of Sustainable Development Goals of the United Nations (United Nations, 2015). However, vegetation is not static but changes dynamically, responding to drivers such as land‐use change and climate change (Erb et al., 2017; Lindner et al., 2010). Thus, predicting future trajectories of vegetation dynamics is highly relevant to decision makers and society.

A key methodological challenge for vegetation modelling lies in addressing ecosystem dynamics across large spatial extents. Key environmental issues such as climate change and biodiversity loss are of global concern (Steffen et al., 2015), and addressing them requires policy responses at national to global levels. Consequently, there is a scale mismatch between the underlying ecological processes of key relevance in this context (e.g. vegetation carbon uptake, species coexistence)—pertaining to the leaf‐ to plant‐scale—and the large scale information demands of decision makers regarding future vegetation dynamics. Scaling, which has been a central issue in ecology for decades (Levin, 1992; Urban, O'Neill, & Shugart, 1987; Wiens, 1989), is a methodological challenge that is thus increasingly important in the context of applying ecological understanding to evidence‐based decision making (Seidl et al., 2013; Seppelt, Lautenbach, & Volk, 2013).

Dynamic global vegetation models (DGVMs) are frequently used for simulating vegetation dynamics across large spatial extents (Snell et al., 2014). This group of models often employs a highly realistic model structure (e.g. leaves or individual trees as entities of the simulation), and assumes that these structures represent the vegetation (and its responses to, for example, climate and management) in a given grid cell (with a typically width of between 10 and 250 km). Recent advances in DGVM development have focused on considerably improving the physiological representation of terrestrial ecosystems, for example, by including nitrogen and phosphorus cycles in addition to the carbon (C) and water cycles typically simulated in such models (Goll et al., 2012; Smith & Azad, 2014).

In contrast, biotic interactions such as seed dispersal and establishment, plant competition, and mortality have received only limited attention in vegetation models applied across large spatial extents (Scheiter, Langan, & Higgins, 2013). Plant mortality, for instance, is often represented by a fixed rate of live biomass loss, disregarding the spatio‐temporal complexity of mortality processes (McDowell et al., 2011). However, biotic interactions and the resulting demographic structure of vegetation are considered to be crucial for a better understanding of C storage (Körner, 2017) and range dynamics (Normand, Zimmermann, Schurr, & Lischke, 2014) in terrestrial ecosystems. Furthermore, the effects of spatial neighbourhood and temporal legacies are important drivers of vegetation structure and biodiversity (Essl et al., 2015; Schertzer, Staver, & Levin, 2015; Thom, Rammer, & Seidl, 2017), yet are rarely considered in current DGVMs.

Improving the simulation of large‐scale vegetation dynamics with regard to biotic interactions (e.g. mortality and demography) and spatio‐temporal controls (e.g. migration and legacy) can build on the extensive experience with stand‐ to landscape‐level vegetation models. Such models simulate vegetation demography as an emergent property of regeneration, growth, and mortality processes (Bugmann, 2001), and frequently account for spatio–temporal interactions in ecosystems (Shifley et al., 2017). However, they usually lack scalability, which limits their application across large spatial extents, and necessitates scaling approaches that are able to capture the drivers of vegetation development at small scales and dynamically scale ecosystem dynamics across spatial domains. Existing approaches for achieving such an upscaling include closed‐form equations (Moorcroft, Hurtt, & Pacala, 2001) and meta‐modelling (Acevedo, Ablan, Urban, & Pamarti, 2001; Cipriotti, Wiegand, Pütz, Bartoloni, & Paruelo, 2016; Urban, 2005).

Recent years have seen the emergence of a new class of algorithms that can loosely be summarized under the moniker of machine learning. These approaches excel at identifying structure in complex, nonlinear data, and generate accurate predictive models (Goodfellow, Bengio, & Courville, 2016). Specifically, Deep Learning is an emerging machine learning technique at the core of recent breakthroughs in computer vision, speech synthesis, autonomous driving, and other fields (LeCun, Bengio, & Hinton, 2015). Yet, such approaches remain underexploited for scaling in ecological modelling to date, despite their inherent potential for generalization.

Here, we introduce the scalable vegetation dynamics (SVD) model, describing a computationally efficient approach to simulate vegetation transitions using deep neural networks (DNNs). The overall aim of the framework is to dynamically simulate vegetation transitions at large spatial scales, and assess their consequences regarding important ecosystem attributes, including but not limited to C storage and biodiversity. A key goal in the design of the framework is generality, that is, it can be applied to a wide variety of systems, and utilize a number of different data sources. In this contribution, we present model tests and applications for forest ecosystems, applying SVD in a meta‐modelling context. Specifically, we demonstrate that (i) SVD assimilates and reproduces the ecosystem responses to climate change as projected by a detailed process‐based model, (ii) trajectories of important forest attributes, such as C storage and biodiversity, can be simulated in a spatio‐temporally explicit manner with SVD, and (iii) the approach is computationally scalable and efficiently simulates vegetation dynamics of large domains (>10^7^ ha) at high spatial grain (10^0^ ha). Code and executable of SVD and example applications are available online at GitHub (https://github.com/SVDmodel/SVD).

## 2 MATERIALS AND METHODS

To simulate vegetation dynamics across large spatial scales SVD integrates a number of conceptual approaches. The general concept employed to achieve high computational efficacy is to classify vegetation into a fine‐grained set of discrete states. Transition pathways and probabilities between states are estimated by a Deep Neural Network (DNN), and are conditional on the prevailing environmental conditions (e.g. climate) as well as the local context (e.g. spatial neighbourhood). The DNN can be trained on empirical data or—as in this contribution—on the results of a detailed process‐based model. Vegetation attributes such as C storage and biodiversity are linked to discrete vegetation states and the residence time of a given cell in that respective state. In this way, SVD not only predicts large‐scale vegetation dynamics, but also allows the quantification of changes in ecosystem attributes associated with vegetation change. The following sections describe these individual components of SVD and their implementation in more detail.

### 2.1 Vegetation transitions

Change is ubiquitous in ecosystems, as they proceed through phases of growth (*r*), conservation (*K*), release (Ω), and reorganization (α) (Holling, 1986). In addition to this intrinsic system dynamics external drivers such as climate change alter vegetation. Vegetation transitions in the *r* and *K* phases, such as the growth and succession of a forest ecosystem, are primarily driven by the prevailing topo‐edaphic and climate conditions. Disturbances (either natural or anthropogenic) are mainly responsible for transitions in the Ω and α stage. Faithfully capturing vegetation transitions is an important focus of ecological research (Hirota, Holmgren, Van Nes, & Scheffer, 2011; Ratajczak, Nippert, & Ocheltree, 2014), and is regarded as a promising strategy to model ecosystem dynamics (Bagchi et al., 2012; Bestelmeyer et al., 2009; Liénard, Gravel, & Strigul, 2015; Yospin et al., 2015). We here define vegetation transitions as a change in the discrete state of an ecosystem. States are described along the dimensions ecosystem structure, composition, and functioning. Indicators were selected to be either widely available for many ecosystems or easily determined from state‐of‐the‐art remote sensing approaches.

*Structure:* A key element of vegetation structure is the vertical utilization of space by plants. Furthermore, canopy height is a good indicator of the developmental stage of long‐lived, sessile organisms. We thus used the dominant height of the vegetation canopy as an indicator for ecosystem structure, here classified into bins with 4 m class width.

*Composition:* The composition of an ecosystem is quantified via the most abundant species characterizing an ecosystem. Systems are described as either being dominated by a single species (i.e., >66% of the biomass in the most abundant species), or by a number of species occurring in mixtures (where each species has >20% of the biomass).

*Functioning:* As processes such as light absorption, transpiration and primary productivity are causally linked to leaves, we used leaf area index as a widely available proxy for ecosystem functioning. Many ecosystem processes saturate with high leaf areas, which is why we limited our classes to three functional states (i.e. densely vegetated [leaf area index LAI > 4], moderately vegetated [2 < LAI ≤ 4], and sparsely vegetated [LAI ≤ 2]).

The potential number of different vegetation states being captured by a full combination of these three indicators is large: Assuming a species pool of 20 species (that all have the potential to dominate species composition), 4 m height classes up to a potential tree height of 100 m, and three classes of LAI, more than 514,000 distinct vegetation states can be distinguished (i.e. 6,597 × 26 × 3). Note that in typical applications only a small fraction of potential states is actually realized. Also, while the scheme presented above is tailored to forest ecosystems, different indicators can be used within the framework presented here (e.g. plant functional types rather than individual species for describing vegetation composition).

### 2.2 Modelling transition probabilities using deep neural networks

In SVD, we use a deep neural network (DNN) approach to model the probabilities and pathways of vegetation transitions. DNNs are a powerful machine learning approach that is able to learn complex relationships in data. It has received considerable attention recently, and is increasingly applied in a wide variety of fields from image and text analysis to medical diagnosis (Esteva et al., 2017; LeCun et al., 2015). DNNs are descendants of artificial neural networks, extending the approach *inter alia* by allowing a substantially larger number of layers (hence the term ‘deep’). Utilizing deeper and therefore more powerful networks became feasible only in recent years, through methodological and technical advances such as improved training algorithms and higher computing power (Goodfellow et al., 2016). DNNs consist of many artificial ‘neurons’ that are organized in layers. Typically, each neuron in a layer is connected to all neurons of the previous layer. A neuron calculates a single output value from its inputs, which subsequently serves as an input for the neurons of the following layer. The input data, provided by the initial layer of the network, is increasingly transformed as it percolates through the network towards the output layer. Note that a single DNN can be designed to handle multiple types of input simultaneously, and can be trained to provide simultaneous information on multiple output variables. The network architecture specifies the detailed layout of a DNN, including the size (number of neurons in each layer and the number of layers) of a DNN, the choice of layer types, the use of regularization techniques, and the activation function for each neuron. Once the network architecture is determined, the parameters in a DNN (i.e. the connection weights between neurons) are estimated iteratively by a learning algorithm during the training of the network. For more detail on DNNs in general, we refer to Goodfellow et al. (2016), Angermueller, and Pärnamaa, Parts, Stegle, and Oliver (2016).

**Figure 1:**
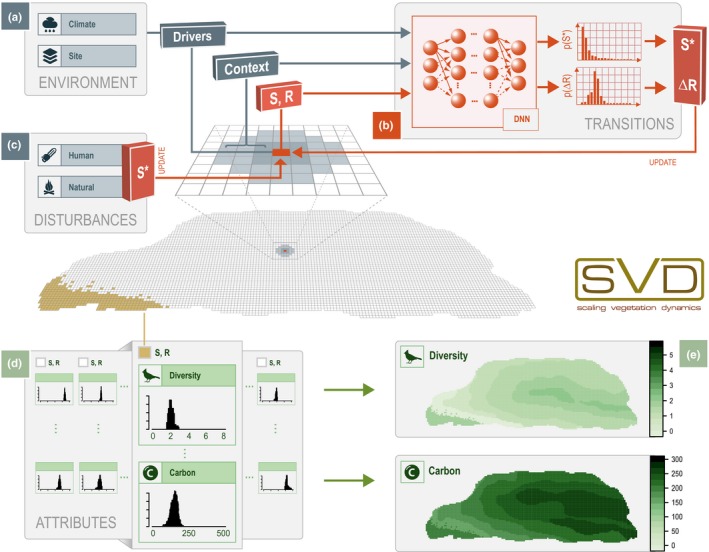
Conceptual view on the scaling vegetation dynamics (SVD) framework. Vegetation transitions on a single cell are estimated by a Deep Neural Network (b) contingent on environmental factors (a), the current vegetation state (*S*), the residence time (*R*) and the spatial context. The model determines transitions by sampling from the DNN‐derived probability distributions for the future state (S*) and the time until state change (Δ*R*). Human and natural disturbances (c) add an abrupt pathway for vegetation transitions and will be described in future work. Density distributions of ecosystem attributes of interest are linked to combinations of *S* × *R* (d). These state‐ and residence time‐specific attribute distributions can subsequently be used to predict changes in the spatial distribution of these attributes based on the simulated vegetation transitions SVD (e)

Here, we trained a DNN to estimate vegetation transitions contingent on the current state (*S*), the residence time (*R*, i.e. the time a cell has already remained in its current state), the spatial neighbourhood, and the prevailing site and climate conditions (Figure 1). The output of the DNN is twofold: First, it predicts a probability distribution describing if and when a transition is likely to happen for a given focal cell within a prediction horizon of 10 years. In other words, the DNN estimates the remaining residence time of a cell within its current state (Δ*R*). Second, the target state of the transition S* is estimated as a probability distribution over possible future states.

As predictors of transitions we used the current state of the vegetation (*S*) and the residence time (*R*) that a cell has been in its current state. In addition, soil depth and soil fertility (time‐invariant) were considered as important local context variables of vegetation development and transition. Climate variables were considered as time‐variant predictors of vegetation transitions. Specifically, we used mean monthly temperature and precipitation for the next 10 years (i.e. the prediction horizon of the DNN) as predictors. The influence of spatial context on transitions was accounted for in two tiers: The local neighbourhood is represented by the eight immediate neighbours of a 100 × 100 m^2^ focal cell. In addition, the intermediate neighbourhood considers a wider spatial influence, and contains cells within a radius of 300 m around a focal cell (Figure S1). In these neighbourhood tiers, species shares are calculated as a proxy for the seed input into the focal cell. In order to consider edge effects, we also included the distance to the edge of the simulated area in the predictors describing the spatial context of a cell. In addition to environmental variables and spatial context, a third group of factors influencing vegetation transitions are disturbances. Both human and natural disturbances hold the potential for rapid transitions between strongly differing vegetation states, signifying the release (Ω), and reorganization (α) phases of vegetation dynamics (Holling, 1986). While we here focus on transitions through the growth (*r*) and conservation (*K*) phases disturbances will be integrated into future versions of SVD (see also Figure 1).

In this contribution, we used high‐resolution process‐based model (PBM) output for training the network, specifically extracting simulated vegetation transitions and their context variables from PBM simulations. By training the DNN on a large number of simulated transitions across a wide variety of ecosystem states and a broad range of environmental conditions, the network learns a ‘meta‐model’ of the underlying PBM. However, SVD is flexible with regard to the network architecture and data used in training, and other variables and datasets as the ones used here could be harnessed in future applications. Further details on modelling transition probabilities using deep neural networks are given in the Supplementary Material S2.

### 2.3 Estimating the effects of transitions on vegetation attributes

Vegetation transitions are modelled as changes between discrete states in SVD, with states defined by indicators of vegetation composition, structure, and functioning. To estimate the effect of these transitions, we track state‐specific attributes of the simulated ecosystem. The underlying notion is that many ecosystem attributes are strongly correlated with the state of the vegetation. We start by assuming that all cells within a certain vegetation state S (e.g. ‘Norway spruce (*Picea abies* (L.) Karst.) dominated, moderately dense forests (2 ˂ LAI ≤ 4) with a canopy height of 20–24 m’) share the same attribute value (e.g. with regard to live tree carbon storage). Knowing the state *S* of every cell within the simulated area is thus sufficient for estimating the ecosystem attribute of interest for the entire simulation area. We subsequently refine this assumption in two important ways. First, in many cases, the residence time *R* in a particular state *S* also influences ecosystem attributes. A forest that recently transited into a certain state *S* (e.g. with a canopy height of just above 20 m) is likely to store less live tree carbon than a forest at the upper bound of the same state (with a canopy height of almost 24 m). We thus differentiate vegetation attributes within states by residence time (*S* × *R*), refining the grain of our state‐based attribute quantification. Second, even after such a refinement, attributes are bound to vary within a given state and residence time category. In order not to overestimate the significance of vegetation transitions for individual attributes it is thus important to consider the full distribution of an attribute within an *S* × *R* category (and not only its central tendency). This allows us to assess whether changes in ecological attributes are in fact significantly different. Consequently, the robustness of attribute changes associated with vegetation transitions can be assessed independently for each attribute in a nonparametric manner.

Conceptually, SVD maintains a database of vegetation attributes that—for each combination of *S* × *R*—provides a density distribution for every ecosystem attribute under investigation. In the current application, this database is populated with values determined from PBM simulations. However, since vegetation transitions and attributes are treated independently in SVD, the attributes database could subsequently be refined by including data from additional sources (e.g. remote sensing, terrestrial forest inventory).

### 2.4 Model implementation

For implementing and applying the SVD framework, a training and an application phase are distinguished. The training phase encompasses the design and training of the DNN and its prerequisite steps (e.g. acquisition and/or generation of training data, populating of the vegetation attribute database). We here utilized the open source machine learning framework TensorFlow (Abadi et al., 2016), which is widely used in deep learning applications. The training phase is an iterative process of refining, training, and testing the DNN, employing the diagnostic tools provided by TensorFlow (see Supplementary Material S2 for details). It concludes with a fully trained DNN for predicting vegetation transitions, which is subsequently used in the application phase. Note that the details of the DNN architecture and training process (e.g. the used data) are encapsulated in the trained model via the network structure and weights, which allows tailoring DNNs for specific applications and/or regions.

In applications, the SVD model serves as the interface for using the trained DNN in dynamic predictions of transitions given new environmental vectors, and scales vegetation dynamics to large spatial extents (Figure 1, see Supplementary Material S1 for technical details). The time step of the model simulations is annual. In each time step, SVD considers the subset of cells for which a transition was estimated based on the 10‐year prediction horizon of the DNN (Δ*R*). It calculates a probability distribution for the new state S* based on DNN predictors, and determines the new state probabilistically by drawing from the distribution. For all cells not subject to vegetation transition during the time step the residence time is advanced by 1 year. At the end of each time step, the ecosystem attributes are updated by querying the attribute database using the newly calculated *S* and *R* for each cell. Attribute values are sampled from the respective density distributions stored in the vegetation attribute database. The current spatial grain of SVD simulations are regular 100 m grid cells, that is, transitions, states, and attributes are calculated at the level of 1 ha.

### 2.5 Model testing and application

To demonstrate the utility of the SVD framework, we conducted a number of simulation experiments, specifically aiming at (a) testing the ability of the SVD to reproduce detailed PBM trajectories during the *r* and *K* phases of vegetation dynamics (i.e. a DNN‐driven meta‐model of a PBM), and (b) demonstrating the applicability of the model to large spatial scales. For testing and comparison to PBM simulations we focused on Kalkalpen National Park (KANP) in the northern Alps in Austria. KANP is a forest landscape of 20,850 ha, containing a range of forest types extending over an elevational gradient from 385 m to 1,963 m (Figure S6 in the Supplementary Material). As reference PBM, we used iLand, the individual‐based landscape and disturbance model (Seidl, Rammer, Scheller, & Spies, 2012). iLand was extensively tested and applied in the region previously (Thom et al., 2017; Thom, Rammer, & Seidl, 2017) and is thus a suitable PBM for meta‐modelling. Starting from today's vegetation at KANP, we simulated natural forest dynamics with iLand over 500 years under four different climate scenarios without consideration of management and disturbances. The simulated vegetation dynamics at a spatial grain of 100 × 100 m^2^ were used to train a DNN. The dataset contained a total of 16.8 Mio training examples distributed over 1,418 distinct vegetation states. We split the data into a training set (three of the four climate scenarios, 75.4% of the examples), and set aside data of one scenario (C3, 24.6% of the examples) as an independent validation set (see Supplementary Material S2 for additional details on KANP, the scenarios considered, as well as the data and training procedure). In order to assess whether the DNN was able to infer additional information from spatial context variables (i.e., the vegetation state of neighbouring cells) we trained DNNs with and without spatial context, and compared their accuracy in predicting vegetation transitions.

Subsequently, we tested the ability of SVD to reproduce the patterns of vegetation development obtained from the PBM in a number of experiments. First, we used SVD to simulate vegetation dynamics at KANP starting from the same initial conditions as the PBM, and assessed whether SVD was able to reproduce the PBM‐derived vegetation development under different climate forcings. In particular, we compared the predicted state of the landscape from SVD to PBM results in terms of composition, structure, and functioning after 100, 300, and 500 simulation years. This test thus examines whether SVD is a good meta‐model of iLand under a variety of climate scenarios. To facilitate the visual interpretation of simulated species composition, we mapped the most frequently occurring compositional types, and binned all remaining types into an ‘other’ category.

The second test evaluated the simulated trajectories of ecosystem attributes, exemplarily focusing on live tree carbon (tC ha^−1^, C) and tree species diversity (exponent of the Shannon α diversity, equaling the first order Hill number, *D*, which can be interpreted as the approximate number of species with equal abundances). We used data from iLand simulations to compile a database of vegetation attributes in SVD, and consequently compared the state‐based attribute predictions of SVD with detailed information on live tree C and tree species diversity as simulated by iLand. As these attributes are an emerging property of detailed physiological and demographic processes in iLand, this second test examined the proposition that they can be reasonably approximated by the *S* × *R* attribute classes of SVD.

Finally, to demonstrate the potential of SVD to scale up vegetation dynamics to large spatial scales we applied the model to a generic landscape of 250 × 1,000 km (2.5 × 10^7^ ha, i.e. approximately the forest area of France) at a spatial grain of 100 × 100 m^2^. For this test, we assumed the gradient in mean annual temperature contained in KANP (i.e., a range of 5.5°C) to vary linearly over the 1,000 km length of the simulated landscape, ordered from the coldest (top) to warmest (bottom) conditions. Starting the simulations from a random initial state (sampled from the 1,418 vegetation states realized in PBM simulations), we simulated vegetation development over 500 years (using a stationary climate representing the conditions of the recent past). We assessed the emerging spatial pattern of species composition for ecological plausibility, i.e. whether a regular pattern over the imposed climate gradient emerged from the randomly initiated vegetation state. This third experiment was thus aimed at evaluating the ability of the model to simulate transient patterns of spatially explicit species change (as relevant e.g. in the context of species migration). In addition, it also aimed at testing the computational performance of SVD when scaling vegetation dynamics across three orders of magnitude (from 2.0 × 10^4^ ha to 2.5 × 10^7^ ha).

## 3 RESULTS

### 3.1 A deep neural network for modelling vegetation transitions

The DNN for estimating vegetation transitions at KANP had nine hidden layers and 1.33 Mio learned connection weights (see Supplementary Material S2 for details). It was well able to learn vegetation transitions and their dependency on current vegetation states and environmental drivers. In an assessment of DNN performance against the independent validation data (i.e. data not used for training the network) it reached a prediction accuracy (i.e. the percentage of correctly predicted classes) of 86.3% for estimating the time until transition (Δ*R*), and 85.7% for the state resulting from a transition S*. Furthermore, the correct new state after a transition (S* as predicted with iLand) was contained in the top three most probable classes predicted by SVD in 97.1% of the cases. The network generalized well, with only slightly decreased classification performance for validation data compared to training data (see Table S3). The prediction accuracy decreased slightly when disregarding the spatial context of a cell (Supplementary Material Figure S4).

### 3.2 Simulating vegetation transitions with SVD

**Figure 2:**
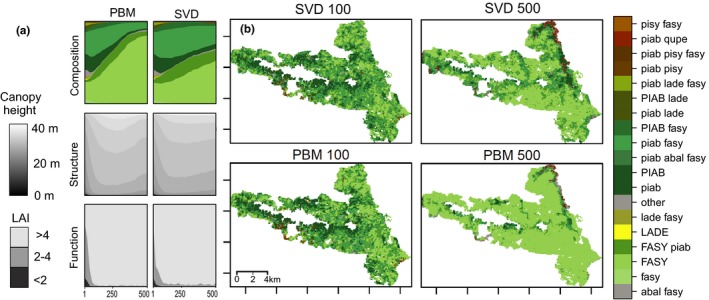
Simulated vegetation transitions in scaling vegetation dynamics (SVD) for Kalkalpen National Park under climate scenario C3 (validation set, data not used for training of the DNN). (a) Distribution of the vegetation state regarding composition, structure and functioning at the landscape scale over 500 years. (b) Spatial distribution of vegetation composition, comparing SVD to the process based model (PBM) iLand after 100 and 500 simulation years. Shown are the 18 most frequently occuring compositional types. The used species codes are ‘piab’ for *Picea abies*, ‘pisy’ for *Pinus sylvestris*, ‘lade’ for *Larix decidua*, ‘fasy’ for *Fagus sylvatica*, and ‘abal’ for *Abies alba*. Dominant species (i.e. >66% of the biomass) are indicated by uppercase species codes

The simulated vegetation dynamics with SVD showed good agreement with the trajectories derived from the PBM iLand (Figure 2, Table 1). Under the climate forcing C3 (validation set, not used for training the DNN), the species composition shifted from an initial Norway spruce dominance to European beech (*Fagus sylvatica* L.) for most parts of the landscape. In the low elevation areas of the landscape (northeastern corner) warm‐adapted species such as oak (*Quercus* ssp.) appeared. These simulated patterns of climate‐induced species change over time were in congruence with PBM simulations. Compared to the PBM, SVD moderately underestimated the share of beech on the landscape after 500 years. However, vegetation structure and functioning developed similarly in SVD and the PBM, displaying a rapid transition to closed forests of tall tree canopies. Results for simulations under the three other climate scenarios showed that SVD responded realistically to different climate signals, reproducing the patterns found in the PBM simulations (Table 1, Supplementary Material Figure S7). In a cell‐wise comparison between SVD and PBM simulations, average accuracies were 0.565, 0.602, and 0.946 for ecosystem composition, structure, and functioning (average values over three points in time (after 100, 300, 500 simulation years) and all climate scenarios, Table 1). When SVD was driven by an alternative DNN neglecting spatial context information in making predictions on vegetation transitions, the resulting spatial pattern was markedly noisier (see Supplementary Material S5).

**Table 1:** Total accuracy (i.e., fraction of 100 m cells correctly classified by SVD) for vegetation composition, structure, and functioning at three points in time. The accuracy for composition was calculated for the 18 most frequently occurring compositional states (see also Figure 2). BL = baseline climate, C1–C3 = scenarios of future climate change. Note that reference data for scenario C3 were not included in training the DNN at the core of SVD

Attribute	Simulation year	Climate scenario			
		BL	C1	C2	C3
Composition	100	0.523	0.514	0.515	0.510
	300	0.617	0.596	0.476	0.469
	500	0.710	0.731	0.572	0.545
Structure	100	0.622	0.623	0.675	0.636
	300	0.565	0.585	0.574	0.538
	500	0.616	0.624	0.576	0.590
Functioning	100	0.908	0.934	0.926	0.955
	300	0.941	0.959	0.943	0.972
	500	0.953	0.957	0.950	0.952

### 3.3 Predicting the response of ecosystem attributes to vegetation transitions

The database of vegetation attributes for KANP contained density distributions for 21,494 combinations of *S* × *R*, and was compiled from 7.7 × 10^7^ data points generated by the process based model iLand. Over all combinations of *S* × *R*, the mean values of live tree carbon ranged from 1.3 to 474.8 tC ha^−1^ (average across all *S* × *R* classes: 150.7 tC ha^−1^), and tree species diversity ranged from 0 (no vegetation) to 8.8 for highly diverse states (average: 2.67). The Supplementary Material provides additional analyses on the distribution of attributes over vegetation composition, structure, and functioning (Figures S9 and S10).

**Figure 3:**
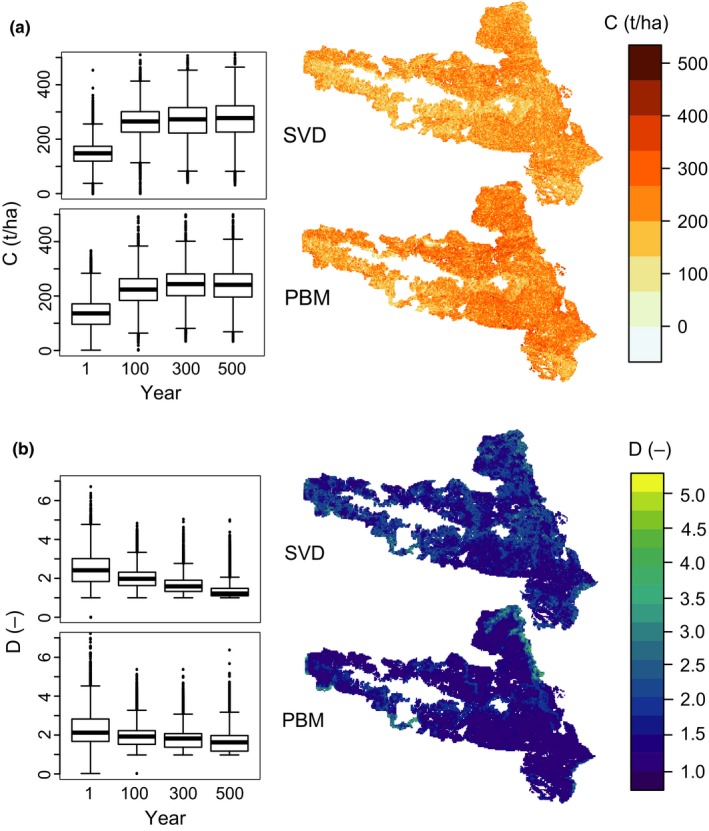
Comparison of the simulated attributes live tree carbon (a) and tree species diversity (b). The boxplots indicate the spatial distribution of the respective attribute over time at the landscape scale. The maps show the spatial distribution of attributes after 500 simulation years. Data is taken from the climate scenario C3 (validation dataset)

Ecosystem attributes predicted by SVD for the climate scenario not used for training (C3) showed a good agreement to PBM values for live tree carbon, and a slightly lower correspondence to reference values for tree diversity (Figure 3). However, SVD correctly predicted the long‐term temporal trend in both attributes, simulating an increase in live tree carbon stocks particularly in the first century of the simulation, and a slow but steady decrease in tree species diversity over time (see Figure S8 for the initial values, and Tables S4 and S5 for results under all climate scenarios).

### 3.4 Upscaling of vegetation dynamics

**Figure 4:**
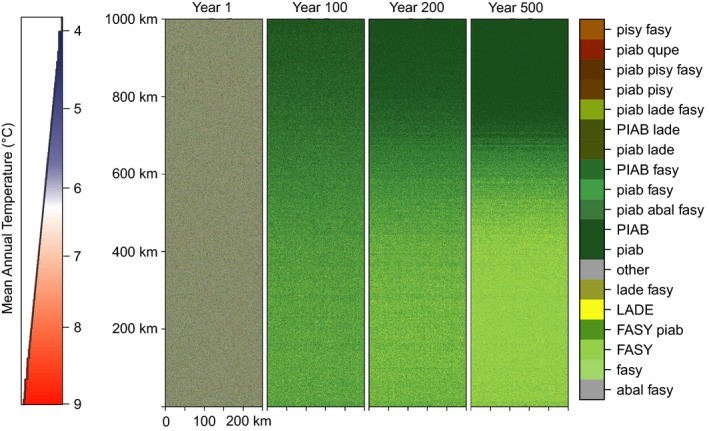
Species composition on a generic landscape with a size of 2.5 × 10^7^ ha with a temperature gradient over 5.5° (left). The simulated vegetation transitions over 500 year from a random initial state (year 1) to clearly separated vegetation zones (year 500). Shown are the 18 most frequent compositional types. The used species codes are ‘piab’ for *Picea abies*, ‘pisy’ for *Pinus sylvestris*, ‘lade’ for *Larix decidua*, ‘fasy’ for *Fagus sylvatica*, and ‘abal’ for *Abies alba*. Dominant species (i.e. >66% of the biomass) are indicated by uppercase species codes

In order to demonstrate the scalability of SVD, we ran a simulation for a generic landscape of 2.5 × 10^7^ ha at a spatial grain of 1 ha over 500 years (Fig. 4). The initially randomly distributed vegetation states clearly separated spatially after 500 simulation years, closely tracking the imposed temperature gradient. The emerging pattern showed a separation in a zone dominated by Norway spruce at the cool end (north), an intermediate zone with mixed spruce—beech forests, and a beech‐dominated zone at the warm end (south). The model was thus well able to realistically separate the major forest types expected for Central Europe (see EEA, 2006) based on climate. This large‐scale application of SVD also proved to be highly computationally efficient: The simulation took less than an hour on a single workstation (i.e. a throughput of more than 4 Mio ha year s^−1^) while consuming only moderate amounts of memory (<3 GB). This underlines that simulating areas at country to continental scale is computationally feasible with SVD.

## 4 DISCUSSION

Scaling has long been a central issue in ecology and remains a challenge for ecological modelling. We here present a novel framework to scale vegetation transitions, combining the discretization of vegetation states with the predictive power of a deep neural network for estimating transition probabilities and pathways. Our approach builds on established concepts used to model vegetation dynamics, particularly the state‐and‐transition modelling approach (Bestelmeyer et al., 2009; Westoby, Walker, & Noy‐Meir, 1989; Yospin et al., 2015). A common element in previous state‐and‐transition models and the SVD approach presented here is the classification of vegetation into discrete states. However, the number of states explicitly considered in traditional state‐and‐transition models is limited (usually in the order of 10s to lower 100s) (Halofsky et al., 2013; Hemstrom, Merzenich, Reger, & Wales, 2007), *inter alia* due to the exponential increase in potential transitions that need to be parameterized. Using a DNN to estimate transition probabilities between a large number of possible states (>514,000 in the example presented here) allowed us to substantially refine the characterization of vegetation, while still exploiting the computational advantages of a finite set of states. A second advancement of SVD over traditional state‐and‐transition models is the explicit consideration of residence time within a vegetation state as a variable influencing the propensity of a transition. This addition considerably improves the realism of simulated vegetation trajectories at the level of an individual grid cell over the use of time‐invariant probabilistic transitions (often troubled by ‘flickering’ between states, or an unrealistically fast/slow progression through a sequence of states).

We found deep neural networks to work well as the engine of our meta‐modelling approach. Specifically, the DNN accurately reproduced the complex responses of an underlying process‐based model, yielding high prediction accuracies for both time until transition (Δ*R*) and the state resulting from a transition (S*). Designing the DNN to consider a prediction horizon (here set to 10 years) rather than estimating transition probabilities from year to year proved an important element of our network architecture, as environmental effects on vegetation can be cumulative over several years (e.g. the impact of drought, Allen, Breshears, & McDowell, 2015). The final DNN was well able to predict situations that were not included in the underlying training data, and thus showed high potential for generalization, which is an important ability in the context of upscaling (Goodfellow et al., 2016; LeCun et al., 2015). The separation of network training and its application in SVD has several advantages. The DNN training procedure is flexible and can therefore be easily tailored to specific applications. Furthermore, the DNN can be continuously improved (e.g. by including new training data) without requiring changes to the SVD model concept. The separation of time‐consuming training and application is also computationally efficient: Compared to the PBM used as a reference (which is already highly optimized for simulation time, see Seidl et al., 2012) the DNN‐driven SVD approach was three to four orders of magnitude faster in simulating vegetation dynamics.

A key limitation of DNNs—and machine learning algorithms in general—is that they require large datasets in order to being able to abstract the underlying relationships. We here used a meta‐modelling approach (Urban, 2005), i.e., training our DNN on extensive simulation results from a detailed PBM. An advantage of meta‐modelling over using empirical information is that also transitions to possible novel future vegetation states (i.e. no‐analog conditions, Williams & Jackson, 2007) are considered. However, the downside of this approach is that our DNN in its current form only encapsulates the processes represented by the underlying PBM, and is trained only for the environmental conditions used in PBM simulations. To increase the robustness of SVD‐predicted vegetation transitions in the future it is thus desirable to increase the database on vegetation transitions available for training the underlying DNN. Such future improvements of the foundation of SVD could, for instance, include simulation results of the same PBM for other ecosystems and environmental conditions (e.g. Seidl, Albrich, Thom, & Rammer, 2018; Silva Pedro, Rammer, & Seidl, 2016). The database could, however, also be extended to include information from different PBMs or increasingly available model comparison experiments (Bugmann et al., 2019; van Oijen et al., 2013), in order to reduce the uncertainty related to the formulation of a single underlying PBM.

An important role of simulation modelling is to make projections on the development of ecosystem attributes that are relevant to managers and policy‐makers. Improving the capacity to model ecosystem attributes in space and time can contribute to the evidence‐based decision making needed for tackling key planetary challenges such as climate change and biodiversity loss (Steffen et al., 2015). We here showed that a combination of ecosystem composition, structure, and functioning can provide a meaningful proxy for quantifying the effect of vegetation transitions on ecosystem attributes of interest. While we here demonstrate our approach for live tree C storage and local tree species diversity, it can easily be extended to a number of other attributes in the future. A particular strength of our approach is that the relationship between a specific vegetation state and an attribute of interest is purely data‐driven, and can consequently be improved by amending the underlying vegetation attribute database, e.g. by assimilating empirical information from forest inventory and analysis data. However, an important limitation of the approach described here is that vegetation states are defined *a priori*, rather than aiming to maximize their explanatory power regarding certain ecosystem attributes (e.g. Peura et al., 2016; Winter & Brambach, 2011). This approach was selected because it grants compatibility with widely available information from remote sensing, which aids the broader applicability of the model. However, when extending SVD to simulate other ecosystem attributes in the future, the ability of the combination of *S* × *R* used in SVD to sufficiently capture differences in these attributes needs to be evaluated. An important tool of inference in this regard is the probabilistic specification of attributes for each *S* × *R*, which allows case‐specific tests on whether a transition between two states results in a significant change in ecosystem attributes.

Our approach to scaling vegetation dynamics complements existing approaches of DGVMs. In comparison to many existing DGVMs, vegetation demography is tracked more explicitly in SVD, which increases the robustness of simulated C stocks (Körner, 2017). Furthermore, the effects of spatial neighbourhood on vegetation transitions are considered explicitly in SVD, which results in more realistic spatio‐temporal trajectories of vegetation change (Snell et al., 2014). We explicitly tested the effect of including spatial context as predictor in our DNN and found that it was important for correctly upscaling patterns of vegetation change (see Supplementary Figure S5), emphasizing that spatial interactions require more attention also at macroecological scales (Rose et al., 2017). However, our approach is not intended to replace existing vegetation modelling approaches but rather to extend the toolbox available for predicting vegetation dynamics. One possible role of our approach could be to synthesize the high level of process detail available in local models, scale up the emerging dynamics, and consistently compare vegetation trajectories to simulations of DGVMs. To date, such comparisons remain strongly limited by the inherent scale mismatch between DGVMs and stand level models (Bugmann et al., 2019). SVD could thus bridge the gap between local (stand to landscape level) models and DGVMs, and foster mutual learning and the advancement of vegetation simulation (Bonan & Doney, 2018).

## 5 CONCLUSIONS

SVD is the—to our knowledge—first vegetation model harnessing deep neural networks. Here, our objective was to demonstrate how to model undisturbed vegetation development (*r* and *K* phases *sensu* Holling (1986)) based on DNNs. However, the model presented here is only a first step, as important drivers of vegetation transitions such as natural disturbances and ecosystem management were not considered here. Given the promising results of this proof‐of‐concept contribution, our future work will focus on including these elements into SVD, and on broadening the extent of its underlying database. We conclude that emerging new technologies such as machine learning and iterative model improvement (Dietze et al., 2018) have the potential to instigate a new wave of model development, and usher in a new era of prediction in ecology.

## AUTHORS’ CONTRIBUTIONS

W.R. and R.S. jointly designed and wrote the paper. W.R. performed the modelling experiments.

## Data Availability

Code and executable of SVD are available online at GitHub (https://github.com/SVDmodel/SVD), and archived at Zenodo (https://doi.org/10.5281/zenodo.2581360). The training data, scripts for DNN training and all files for running the demonstration example for SVD are available at GitHub (https://github.com/SVDmodel/models) and at Zenodo (https://doi.org/10.5281/zenodo.2581371).
