# Supplementary material for: A scalable model of vegetation transitions using deep neural networks
Source: Methods Ecol Evol. 2019 Mar 21;10(6):879–90. doi: 10.1111/2041-210X.13171 (PMC6582592; doi:10.1111/2041-210X.13171)
Supplement: Supplementary file 1 [file MEE3-10-879-s001.pdf]

Supplementary Material: A scalable model of vegetation transitions using deep neural networks

Werner Rammer, Rupert Seidl

S1: A scalable model of vegetation transitions

SVD is a model for the simulation of vegetation dynamics across large spatial extents. The core component is a deep neural network (DNN) which predicts the transitions between vegetation states for each simulated cell (cell size currently set to 100m). The DNN itself can be trained with data from different sources, for instance from simulation modeling or remote sensing. Section S2 provides more details on the specific setup and training of the network used in this study. For the description in S1 we assume that a fully trained network is already available.

The SVD model is a standalone software that integrates the Deep Learning framework TensorFlow (Abadi *et al.* 2016) for DNN inference, i.e. the process of applying a trained model with new data. The core model is programmed in C++, which not only provides high flexibility and performance but also allows a full technical integration of the core libraries of TensorFlow into the framework. The model is designed with a particular focus on performance: it makes heavy use of parallel processing, facilitating the multicore architecture of modern CPUs and offloads the DNN inference to a graphical processing unit (GPU) whenever possible. In addition, the model is memory efficient, as the state of a simulated cell is fully described by only a few values (currently 16 bytes). Consequently, SVD can simulate large areas >10^7 ha on standard workstations, processing millions of ha per second. SVD is published under an open source license and available at GitHub (https://github.com/SVDmodel/SVD).

Table S1 summarizes the data required for a typical SVD application. Raster based spatial inputs are used to define the configuration of the landscape. The initial state of the vegetation (State *S*, residence time *R*) for each cell can be derived from different sources (e.g., remote sensing) but needs to comply with the state scheme used for DNN training. Local conditions (e.g., soil data) are accounted for via time-invariant variables (e.g., soil properties, SSC). An important driver for ecosystem dynamics is climate: here SVD utilizes a time series of climate indicators (CLIM) that may differ in type and temporal resolution. For instance, broadly available monthly mean values of temperature and precipitation or derived BIOCLIM indicators may be used. Multiple 100 m cells can share the same climate data (i.e. assuming homogeneous conditions).

Table S1. Overview over types of input data used by the SVD model.

S, R	Vegetation state and residence time (initial state, gridded)
SSC	Gridded variables describing the local site conditions, e.g. soil properties
CLIM	Climate data; multiple time series, each series with a record per year
DNN	A trained deep neural network (see S2)
VAD	Vegetation attribute database; density distributions of a number of vegetation attributes of interest (e.g., live tree carbon) for all combinations of states and residence times

What follows is a brief description of the steps during the simulation of a single year in SVD. Conceptually, SVD consists of two parts which are executed in parallel. The first part (*core* model) keeps track of the simulated landscape, provides the input data for DNN inference, and processes the DNN results. The second part (*DNN*) receives data from the *core* model, runs the DNN inference, and returns predicted changes. The design reflects the fact that DNN inference is most efficiently executed in "batches" on a GPU, i.e. a number of cells are evaluated in parallel in a single operation (with the *batch size* being a model parameter, e.g., 1000). When a new year starts, the *core* model evaluates for each cell whether an update is necessary, i.e. if a state transition has been scheduled for the cell in the current year in the model predictions over the ten year prediction horizon. For cells that need an update, the model collects the necessary information for DNN inference (e.g., the climate data for the upcoming ten years, and the spatial context based on the state of the neighboring cells). Whenever a batch is full (or the landscape is fully traversed), the data is sent to the *DNN*. Based on the predictions of the *DNN*, the *core* model schedules future updates (e.g., one cell might transition to a certain state in four years, while another cell might remain in the same state for the next ten years). After all cells have been processed, the scheduled changes for the current year are applied, and the residence time (R) of each cell is increased. Whenever the *DNN* model receives a batch of cells, it runs the neural network inference using TensorFlow, and returns a probability distribution over the future state S^* and time-until-state-change (ΔR) for each cell. The model first chooses ΔR probabilistically; if this calculation results in a prediction of "no change" for the time period (as indicated by predicting ΔR to be >=10 years) the vegetation state remains unaltered. Otherwise, a future state is selected from the probability distribution of the top N classes (excluding the current state), i.e. the states for which the DNN predicted the highest probabilities (N is currently 10). By choosing from the top N classes very unlikely (and potentially unrealistic) state changes are excluded and the robustness of the prediction is increased. Finally, the processed batch is returned to the *core* model.

At the end of a time step, the new state for each simulated cell is available, as indicated by updated values for of S and R. Consequently, updated values of ecosystem attributes can be queried from the vegetation attributes database.

The outputs of SVD include wall-to-wall maps for S and R for any given time step as well as results for ecosystem attributes derived from VAD for these states. Since the attributes data are probabilistic for each state, several options are available: If the central tendency is of particular interest the mean/ median of the attribute distribution can be assigned for each combination of $S \times R$. Uncertainties can be assessed via the standard deviation or percentile ranges of the underlying distributions in the VAD.

S2: Deep neural network training

Training data

This section describes the training of the DNN used in the application described in the main text. We used the individual based forest landscape and disturbance model iLand (Seidl *et al.* 2012) to generate training data for the DNN. We applied iLand to Kalkalpen National Park (KANP) in the Austrian Alps. The landscape with a size of 20,850 ha is located in the northern front range of the Alps (N47.47°, E14.22°) and ranges from 385 m to 1,963 m a.s.l. It encompasses three of the most important forest types of Central Europe, that is, European beech (*Fagus sylvatica* (L.)) forests, Norway spruce (*Picea abies* (L.) Karst.) forests, and mixed forests of Norway spruce, silver fir (*Abies alba* (Mill.)), and European beech. More details to the study landscape can be found in Thom *et al.* (2016) and Thom, Rammer & Seidl (2016, 2017). In order to generate training data for the DNN, we simulated forest dynamics at KANP starting from the current landscape composition (2013), and running iLand over 500 years without consideration of natural disturbances and management. For initial conditions of the vegetation see Figures S6 and S8. The simulations included four climate scenarios, namely a baseline scenario of historic climate (resampled from the period 1950-2010, BL), and three SRES A1B scenarios including CNRM-RM4.5 driven by the global climate models (GCM) ARPEGE (C1), and MPI-REMO (C2), as well as ICTP-RegCM3 driven by the GCM ECHAM5 (C3). A stabilization of the climate after 2100 was assumed and the climate data for the years 2100-2500 were sampled from the period 2080-2100 of the respective scenario (see Thom, Rammer & Seidl (2017) for details). The size of the simulated species pool was 31. The outputs of the simulation included detailed information on vegetation transitions at a spatial grain of 100m. Specifically we deduced the time, vegetation state, and spatial neighborhood of cells for which iLand predicted vegetation transitions, and also recorded when no transition was simulated over a ten year period. The spatial context was calculated for both the local and the intermediate neighborhood (Figure S1), and was defined as the average share of each species in these neighborhoods. In addition, we derived annual values for selected ecosystem attributes for each from iLand. The attributes used in this study were live tree carbon (C) and D, the exponent of the Shannon index for alpha-diversity (based on basal area shares of tree species on a given cell).

Training data for the DNN was generated from the raw simulation output of iLand (Table S2). The simulations yielded a total of 16.91 Mio training examples describing 3541 unique vegetation states as per the SVD state classification. In order to provide a minimum number of examples for each state, we removed all states that occurred with a frequency of less than 0.0001%. The thus derived set included 16.82 Mio examples in 1418 unique states, containing 142 compositional, 14 structural, and 3 functional classes. 16.7% of the examples contained

state transitions within a 10 year prediction horizon. We split the data into a training set (12.68 Mio examples) and an independent validation set (4.15 Mio examples) based on climate scenarios, with the ICTP scenario (C3) constituting the validation data set. The validation set was not used in training the DNN and was applied to test the generalization performance of the DNN. As the DNN was trained on data from a PBM, for which all drivers are known (e.g., from sensitivity analyses – see Seidl *et al.* 2012), we here did not conduct a formal variable selection.

Table S2. The variables contained in each training example. Each transition in the training data is described by a total of 307 numerical input variables, and two response variables.

**Predictors (input data)**	
S	Current state
R	Residence time, the number of years the cell is already in state S
NB	Spatial context; the relative share of the available species (N=31) for the local and intermediate neighborhood (62 values) (see Figure S1)
SSC	Two indicators for static site conditions: fertility rating (plant available nitrogen in $kg/ha*yr^{-1}$) and soil depth (m).
$CLIM_{10}$	Monthly mean values for temperature and precipitation for 10 years, with 12x2x10=240 values per example
DES	Distance to the closest source for external seed input outside of the simulated area (value of 1 for minimum distance, and 0 for a distance >400m)
**Response variables (labels)**	
S*	Next vegetation state (can be equal to S)
ΔR	Time until state change (or 10 if S=S*)

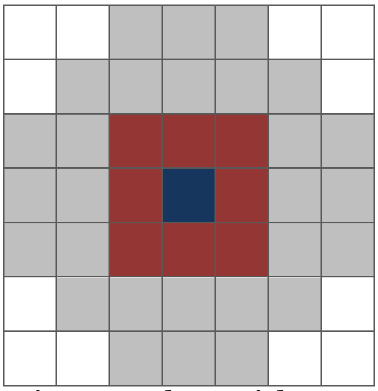

Figure S1. The spatial context of a focal cell (blue) is determined by its local Moore neighborhood (red) and by an intermediate neighborhood (grey). The intermediate neighborhood includes areas within 300m of the focal cell, which approximately resembles typical seeding distances of tree species. Note that the relative strength of the two types of neighborhoods is not set a priori, but is learned by the DNN.

Network structure

We used TensorFlow (Abadi *et al.* 2016) and the top-level Python library Keras (https://keras.io/) for defining the DNN architecture as well as for network training. The architecture of the DNN was a feed forward neural network with 1.33 Mio trainable parameters that integrated concepts from natural language processing (Figure S2). The DNN merged different types of inputs (see Table S2) and jointly trained state transitions (S^*) and time until transition (ΔR) as response variables. The final layer for both response variables was a Softmax classification layer (i.e., providing a probability distribution over 10 and 1418 classes, respectively). The initial Embedding layer (Gal & Ghahramani 2015; Goodfellow, Bengio & Courville 2016) transforms numeric state labels (that carry no information per se) into a N-dimensional vector in which each state is represented as N scalars. During the training, the network learns to map semantically similar states to similar values in embedding space (see Fig S3 for a visualization using the t-SNE algorithm (Maaten & Hinton 2008)). Following the notion that the response to a given climate forcing remains consistent over time, we used a "TimeDistributed" layer that applies the same weights for each year in the climate input data. To decrease the generalization error of the network, we included Dropout layers.

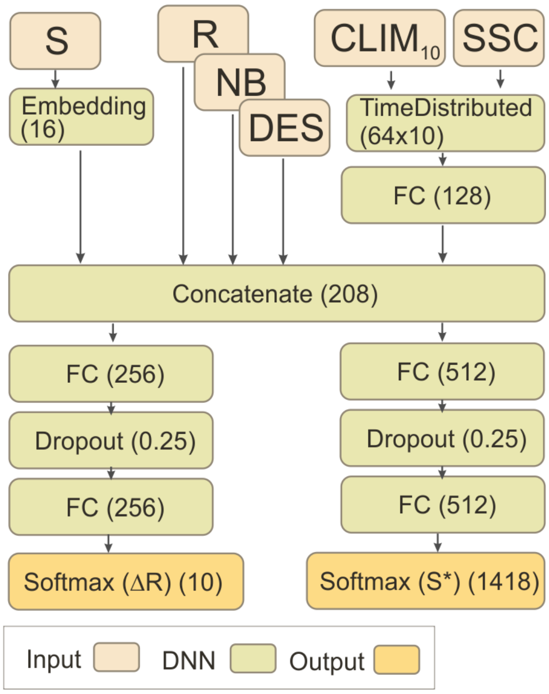

Figure S2. Structure of the trained DNN. FC=fully connected layer. Numbers in parenthesis indicate the number of neurons in each layer. State and climate inputs are processed separately and merged with all other inputs to a single layer (Concatenate layer). From this layer two separate branches for ΔR and S^* lead to the two final Softmax classification layer.

We used categorical cross-entropy as the loss function for both output layers and calculated the total loss as a weighted sum (state: 0.66, time: 0.33). After evaluating different activation functions, we selected the Exponential Linear Unit (Clevert, Unterthiner & Hochreiter 2015)

which showed slightly better performance than rectified linear units or self-normalizing linear units (Klambauer *et al.* 2017).

We use the ADAM optimizer (Kingma & Ba 2014) and a simple scheme to reduce the learning rate after three consecutive epochs without progress by a factor of 0.5 from 0.001 to 0.00001. The training of the final network took approximately three hours on a single workstation (Intel i5-6600 CPU, Nvidia GTX-1070 GPU). The final network structure and parameters were stored for later use in SVD applications.

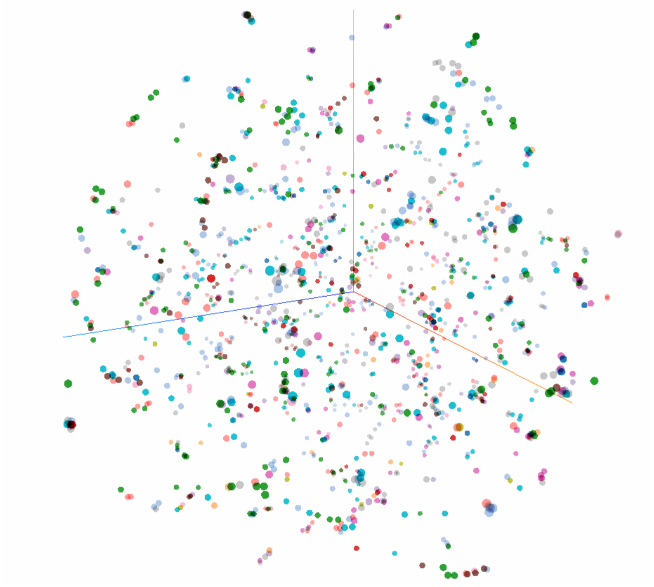

Figure S3. Visualization of the state space learned by the Embedding layer (t-SNE algorithm). t-SNE is a dimension reduction technique (the original embedded space has 16 dimensions). Each dot indicates a single state (N=1418), colors denote the "structure" dimension (height class).

Results of the training and experiments

The DNN was able to predict vegetation transitions accurately over a wide range of climatic (e.g., mean annual temperatures from 3.5 to 13° Celsius) and edaphic conditions. The achieved classification accuracy (categorical cross entropy) was 0.86 for both the future state and time until state transition. More insightful than the raw accuracy information is top-K accuracy: This is the fraction of examples for which the network predicted either the correct label ("Correct") or the correct label was within the top K predicted classes (Table S3). In this context it is important to note that the simulated vegetation transitions include a degree of random variation due to the stochasticity of the underlying PBM. Nonetheless, the final DNN was very well able to reproduce PBM-simulated transition pathways: For 97.1% of the examples in the validation data set (which were not used for DNN training), the "correct" target state was in the top-3 predicted states (from 1418 possible states). The prediction of ΔR

was more challenging, most likely because the exact time of a state transition is less predictable than the eventual target state. Nonetheless, in 91.2% of the cases, the correct year was in the top-3 predicted years.

Table S3. Prediction accuracy of the DNN for the training and the validation data sets. An example is considered as correct, when the predicted class exactly equals the correct label. Top N considers examples where the true label was within the top N predictions.

	Training set		Validation set	
Measure	State (S^*)	Time until transition (ΔR)	State (S^*)	Time until transition (ΔR)
Correct	0.846	0.841	0.857	0.863
Top 3	0.974	0.902	0.971	0.912
Top 5	0.993	0.940	0.991	0.943
Top 10	0.998	1.0	0.997	1.0

Effect of spatial context

In the underlying process based model iLand the simulated transitions depend not only on the vegetation state and the environmental conditions experienced on a given cell, but are also influenced by neighboring cells, e.g. due to an influx of seeds. SVD incorporates spatial context information: For example, the DNN might learn that a transition to a state with higher oak share is more likely, if oak is already present in neighboring cells. We tested the relevance of spatial context by training DNN variants with and without spatial context information, and compared their performance regarding prediction accuracy metrics. The version with spatial context used all available information as described in the previous section (i.e., the species distribution in the local and intermediate neighborhood (Figure S1) as well as the distance to the nearest seed source outside of the boundary of the simulated area), while the version without spatial context lacked both types of information. Figure S4 shows that the DNN was able to extract meaningful information from the spatial context information, and that including this data improved the DNN predictions.

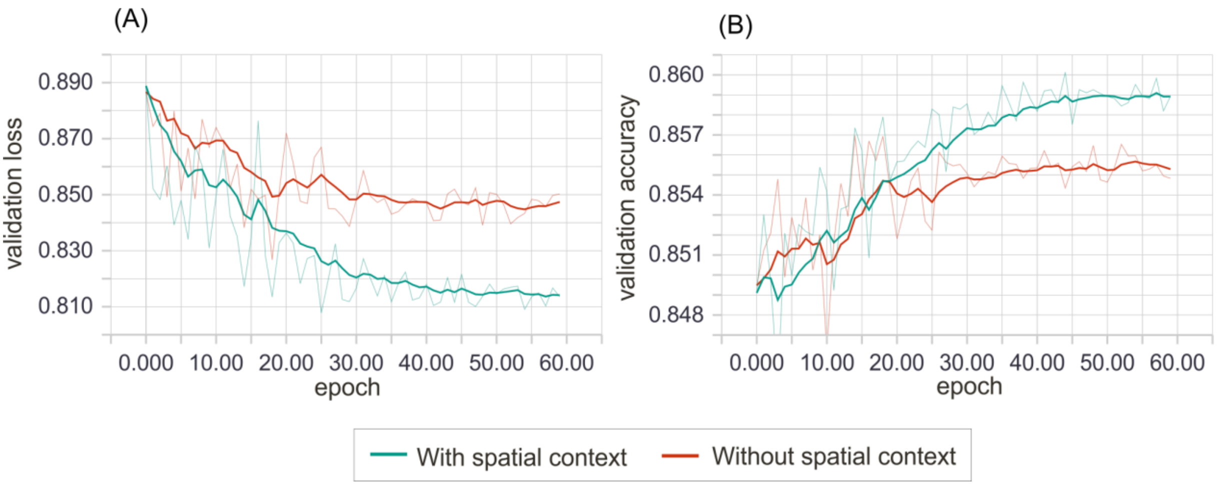

Figure S4. DNN training performance for models with and without spatial context information. A: total loss on the validation data set (lower is better), B: accuracy relative to the validation data set for the classification of the future state (higher is better). Classification performance improved when spatial context information was available during training.

We also analyzed whether disregarding spatial context information translates into different vegetation patterns in the dynamic simulations with SVD. Figure S5 indicates that while the broad spatial patterns (e.g., areas dominated by beech or Norway spruce) persisted in both variants, the results without spatial context showed much higher local variation/ noise.

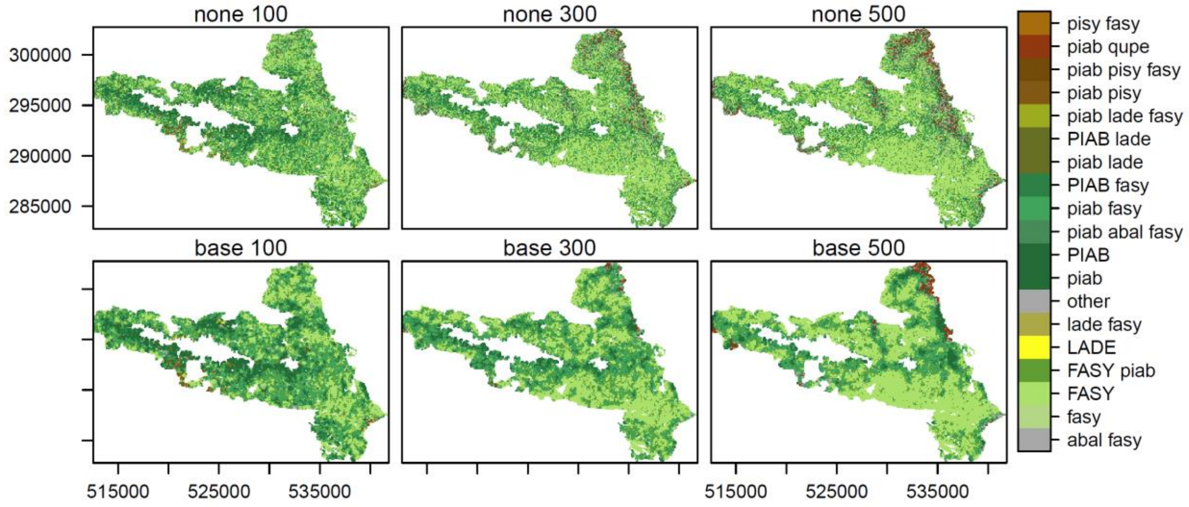

Figure S5. SVD results for species composition using a DNN that was trained without spatial context information (upper panel), compared to a run with full spatial context (lower panel). Shown are the 19 most frequent compositional states (including a category for all "other" states). Uppercase species codes denote states with dominance of a species (>66% of the biomass), lowercase species indicate admixed species (20% < biomass share ≤ 66%). Species codes: "piab": *Picea abies*, "pisy": Pinus *sylvestris*, "lade": *Larix decidua*, "fasy": Fagus sylvatica, "abal": *Abies alba*. Results are shown after 100, 300, and 500 years for the validation climate scenario C3. See also Figure S7.

S3: Additional figures and tables

Kalkalpen National Park

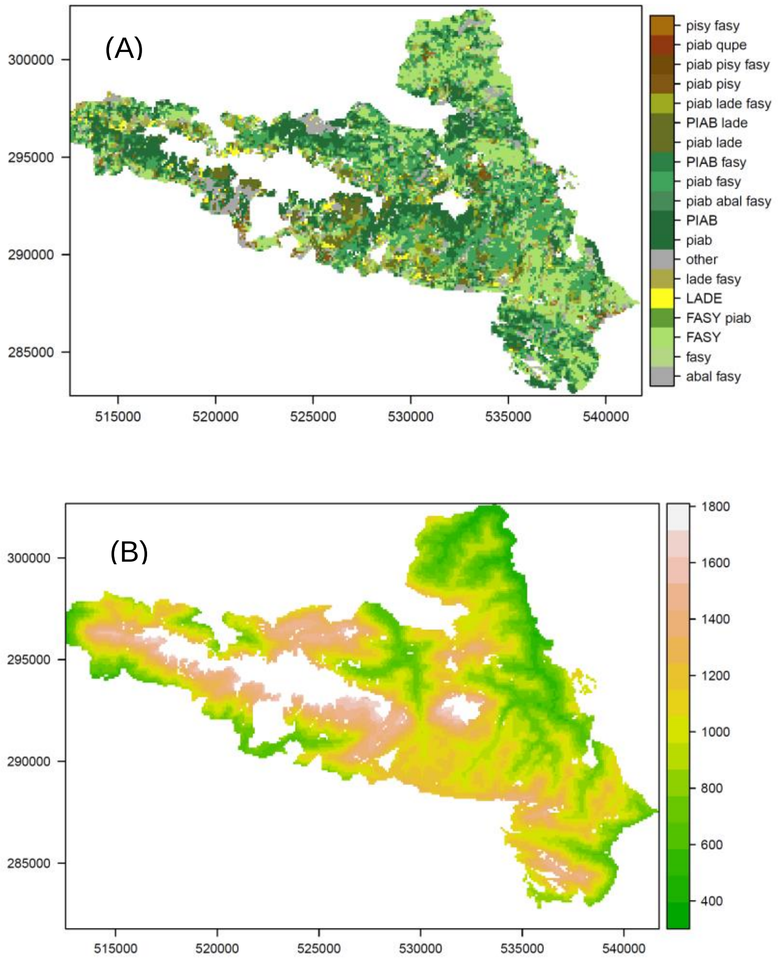

S6. (A) Initial species composition on the landscape. Shown are the 19 most frequent compositional states (including a category for all "other" states). Uppercase species codes denote states with dominance of a species (>66% of the biomass), lowercase species indicate admixed species (20% < biomass share ≤ 66%). Species codes: "piab": *Picea abies*, "pisy": Pinus *sylvestris*, "lade": *Larix decidua*, "fasy": Fagus sylvatica, "abal": *Abies alba*. (B) Elevation above sea level of the landscape (m).

Simulating vegetation transitions

The following figures compare the simulated species composition in SVD with the results of the process-based model iLand for all four climate scenarios considered.

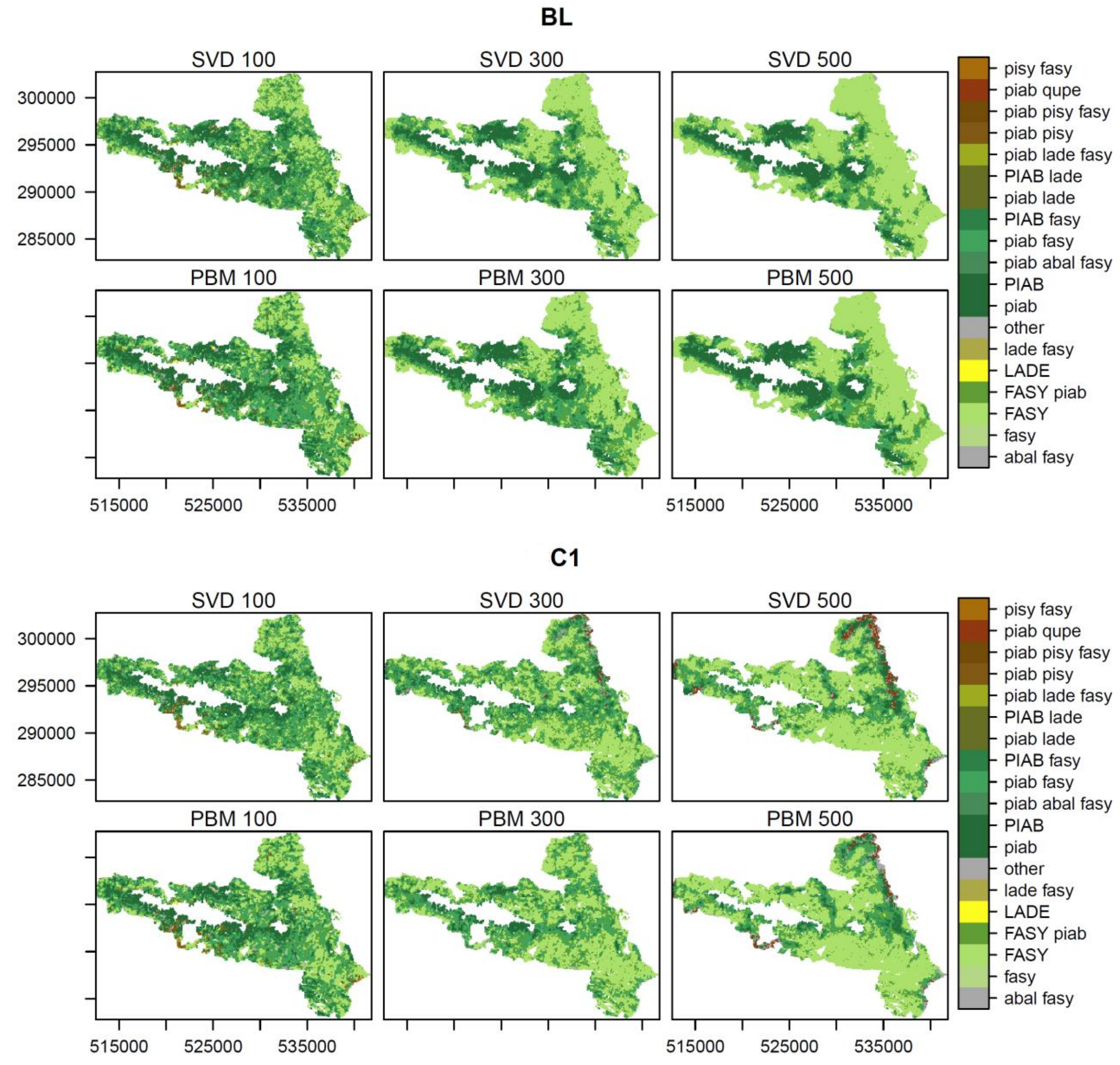

Figure S7. **(Continued on next page)**

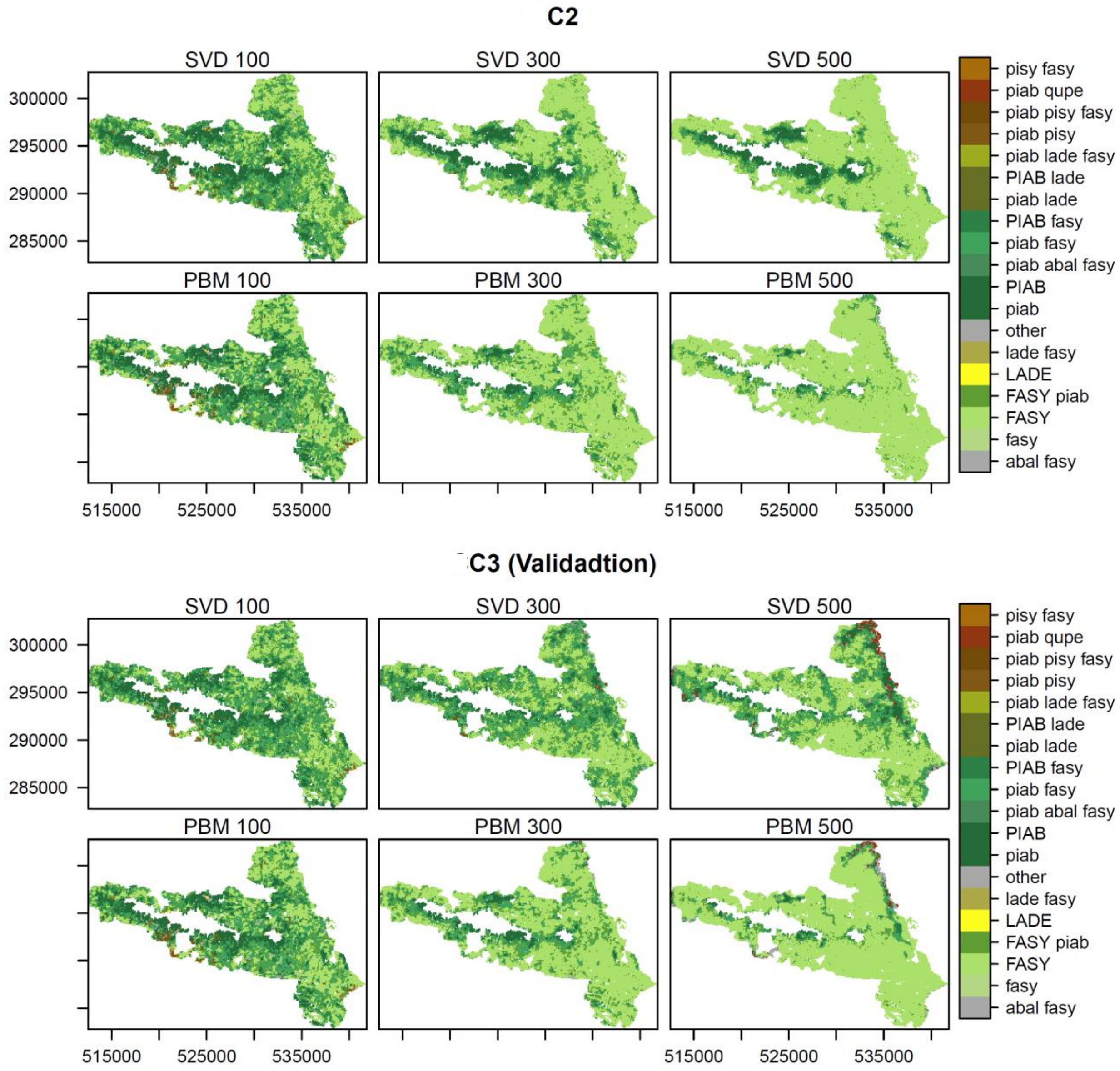

Figure S7. Comparison of SVD simulations to the process-based-model iLand for two climate scenarios. Shown are the 19 most frequent compositional states (including a category for all "other" states). Uppercase species codes denote states with dominance of a species (>66% of the biomass), lowercase species indicate admixed species (20% < biomass share ≤ 66%). Species codes: "piab": ***Picea abies*****, "pisy": Pinus** ***sylvestris*****, "lade":** ***Larix decidua*****, "fasy": Fagus sylvatica, "abal":** ***Abies alba.*** **Results are shown after 100, 300, and 500 years for the four climate change scenarios. C3 was used as the validation data set.**

Ecosystem attributes

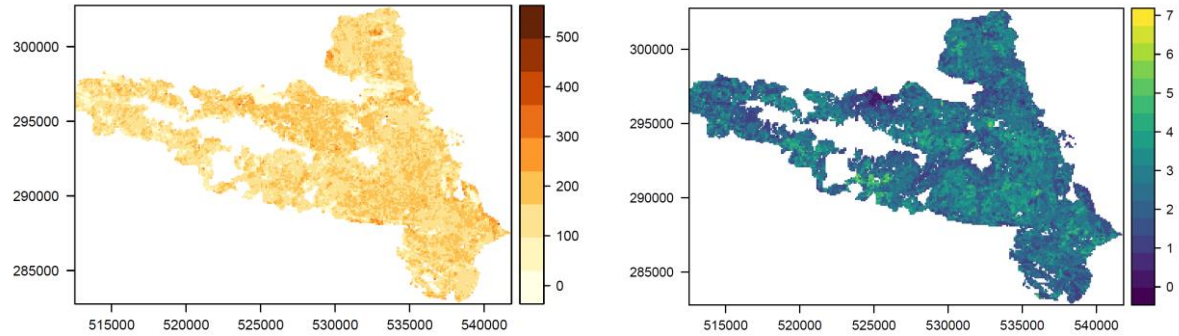

Figure S8. Initial values for live tree Carbon (left, tC ha^{-1}) and the exponent of the Shannon tree species diversity index D (right, dimensionless).

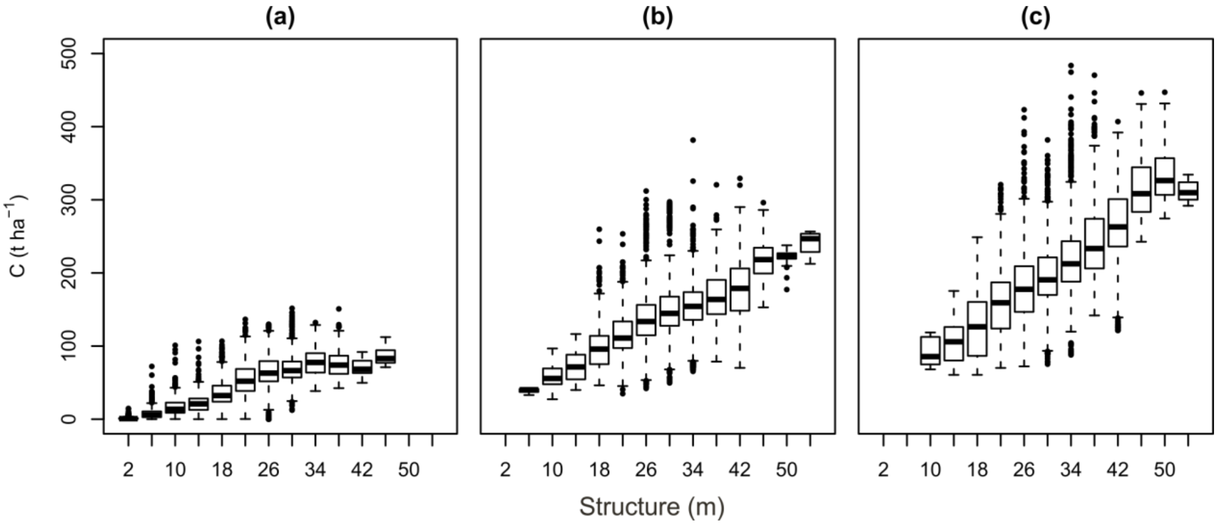

Figure S9. Association of live tree carbon (t ha^{-1}) to structural vegetation states in the three functional classes (a: sparse, b: moderate, c: dense vegetation).

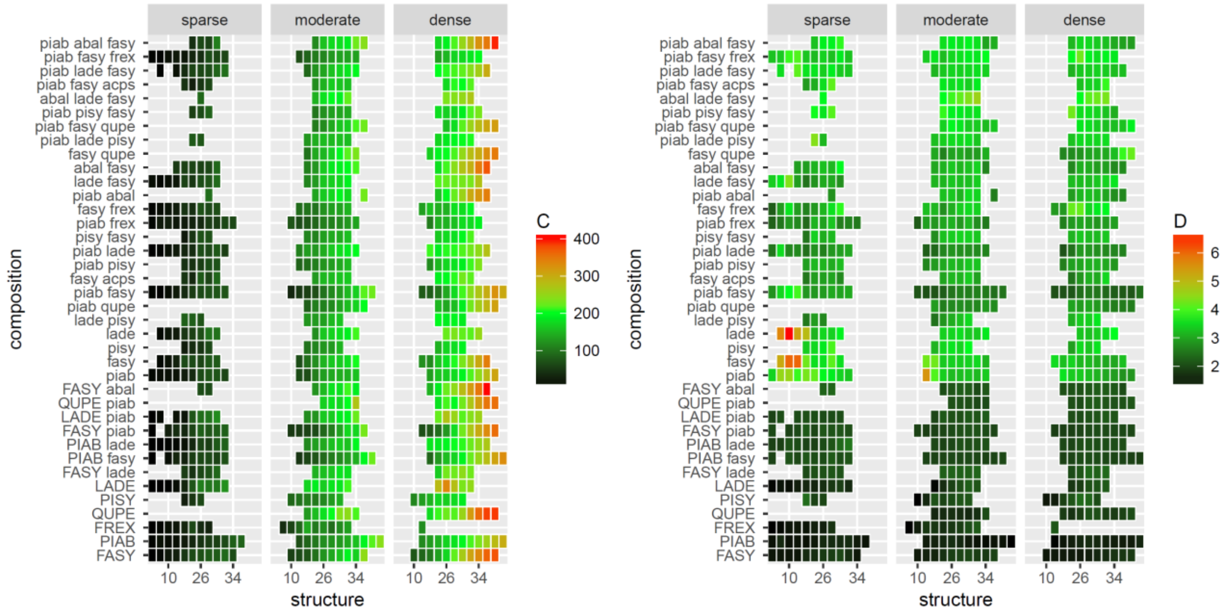

Figure S10. Mean attribute values for live tree carbon (t ha^{-1}, left) and exponent of the shannon index (dim., right). Shown are the 38 most frequent compositional states (out of 142 realized states in total) over structural states (tree height classes, m) and functional states (sparse, moderate, and densely vegetated). Uppercase species codes denote dominant species (i.e., >66% of biomass), lowercase codes indicate admixed species (20% < biomass share ≤ 66%). Compositional states are ordered from those dominated by a single species (bottom) to classes with three admixed species (top).

Table S4. Live tree carbon (C, t ha^{-1}) as simulated with the process-based model iLand (PBM) and SVD for six points in time and four climate scenarios (BL, C1, C2, C3, data from C3 was not used for the training of the DNN applied in SVD). Values for SVD are derived from N=100 landscape realizations derived by sampling from the distribution of indicator values in VAD. Given are the median and the range between the 5th and 95th percentile (in parenthesis)

	BL		C1		C2		C3	
Year	SVD	PBM	SVD	PBM	SVD	PBM	SVD	PBM
1	133.0 (123.9-141.9)	142.0	133.0 (123.9-141.9)	142.0	133.0 (123.9-141.9)	142.0	133.0 (123.9-141.9)	142.0
100	222.5 (214.3-229.6)	234.5	225.3 (217.3-232.8)	250.4	220.9 (212.5-228.4)	227.3	222.7 (214.1-230.8)	261.5
200	237.5 (230.1-246.7)	239.9	240.1 (231.0-249.0)	252.5	234.9 (227.1-243.5)	229.2	233.7 (226.1-242.8)	269.9
300	241.4 (233.7-251.5)	226.2	242.7 (232.1-250.8)	240.9	240.6 (233.9-249.7)	217.6	241.0 (233.9-249.4)	266.7
400	243.8 (234.4-253.1)	224.9	238.4 (226.5-248.5)	228.3	242.4 (234.0-251.6)	217.1	240.5 (232.4-249.2)	271.3
500	241.6 (231.9-251.6)	228.8	236.6 (223.8-247.0)	233.6	239.5 (230.2-249.2)	224.4	238.8 (229.8-248.2)	270.9

Table S5. The exponent of the Shannon index of tree species diversity (D, dim.) as simulated with the process-based model iLand (PBM) and SVD for six points in time and four climate scenarios (BL, C1, C2, C3, data from C3 was not used for training of the DNN applied in SVD). Values for SVD are derived from N=100 landscape realizations derived by sampling from the distribution of indicator values in VAD. Given are the median and the range between the 5th and 95th percentile (in parenthesis)

	BL		C1		C2		C3	
Year	SVD	PBM	SVD	PBM	SVD	PBM	SVD	PBM
1	2.29 (2.17-2.44)	2.46	2.29 (2.17-2.44)	2.46	2.29 (2.17-2.44)	2.46	2.29 (2.17-2.44)	2.46
100	1.89 (1.84-1.98)	2.01	1.87 (1.82-1.95)	2.01	1.94 (1.88-2.03)	2.08	1.93 (1.88-2.03)	2.01
200	1.73 (1.67-1.8)	1.77	1.73 (1.67-1.80)	1.82	1.87 (1.81-1.95)	1.89	1.86 (1.80-1.94)	1.78
300	1.64 (1.58-1.70)	1.65	1.60 (1.54-1.67)	1.65	1.82 (1.77-1.88)	1.83	1.80 (1.75-1.87)	1.64
400	1.57 (1.51-1.64)	1.55	1.50 (1.44-1.59)	1.48	1.75 (1.70-1.82)	1.78	1.75 (1.7-1.83)	1.50
500	1.52 (1.46-1.60)	1.47	1.43 (1.37-1.54)	1.37	1.69 (1.63-1.78)	1.71	1.69 (1.63-1.77)	1.38

References

Abadi, M., Barham, P., Chen, J., Chen, Z., Davis, A., Dean, J., Devin, M., Ghemawat, S., Irving, G., Isard, M., Kudlur, M., Levenberg, J., Monga, R., Moore, S., Murray, D.G., Steiner, B., Tucker, P., Vasudevan, V., Warden, P., Wicke, M., Yu, Y. & Zheng, X. (2016). TensorFlow: A system for large-scale machine learning. *Osdi '16 Proceedings*.

Clevert, D.-A., Unterthiner, T. & Hochreiter, S. (2015). Fast and Accurate Deep Network Learning by Exponential Linear Units (ELUs). 1–14.

Gal, Y. & Ghahramani, Z. (2015). A Theoretically Grounded Application of Dropout in Recurrent Neural Networks.

Goodfellow, I., Bengio, Y. & Courville, A. (2016). *Deep Learning*. MIT Press.

Kingma, D.P. & Ba, J. (2014). Adam: A Method for Stochastic Optimization.

Klambauer, G., Unterthiner, T., Mayr, A. & Hochreiter, S. (2017). Self-Normalizing Neural Networks.

Maaten, L. Van Der & Hinton, G. (2008). Visualizing Data using t-SNE. *Journal of Machine Learning Research 1*, **620**, 267–84.

Seidl, R., Rammer, W., Scheller, R.M. & Spies, T. a. (2012). An individual-based process model to simulate landscape-scale forest ecosystem dynamics. *Ecological Modelling*, **231**, 87–100.

Thom, D., Rammer, W., Dirnböck, T., Müller, J., Kobler, J., Katzensteiner, K., Helm, N. & Seidl, R. (2016). The impacts of climate change and disturbance on spatio-temporal trajectories of biodiversity in a temperate forest landscape. *Journal of Applied Ecology*, in revision.

Thom, D., Rammer, W. & Seidl, R. (2016). Disturbances catalyze the adaptation of forest ecosystems to changing climate conditions. *Global Change Biology*, 1–14.

Thom, D., Rammer, W. & Seidl, R. (2017). The impact of future forest dynamics on climate: interactive effects of changing vegetation and disturbance regimes. *Ecological Monographs*, **87**, 665–684.
